# Chronic time pressure as a predictor of symptoms of depression, anxiety and stress

**DOI:** 10.1186/s40359-025-03654-4

**Published:** 2025-11-24

**Authors:** Ruth Ogden, Christine Schoetensack, Tereza Klegr, José Vicente Pestana, Rafael Valenzuela, Katarzyna Goncikowska, Georgina Giner-Domínguez, Julie Papastamatelou, Sébastien Chappuis, Mónica Fernández Boente, Quentin Meteier, Vanda Černohorská, Nuria Codina, Chantal Martin-Söelch, Marc Wittmann, Joanna Witowska

**Affiliations:** 1 School of Psychology, Liverpool John Moores University, 3 Byrom Street, Liverpool, L3 3AF UK; 2 Institute of Philosophy, Czech Academy of Sciences, Jilska 1, Prague 1, 110 00 Czech Republic; 3 Department of Social Psychology and Quantitative Psychology, University of Barcelona, Campus Mundet, Ponent Building, 4Th Floor. Passeig de La Vall d’Hebron 171, Barcelona, 08035 Spain; 4 The Institute of Psychology, Polish Academy of Sciences, Stefana Jaracza 1, Warsaw, 00-378 Poland; 5 Institute for Frontier Areas of Psychology and Mental Health, Wilhelmstraße 3a, Freiburg, 79098 Germany; 6 Clinical and Health Psychology Unit, Department of Psychology, University of Fribourg, Fribourg, 1700 Switzerland; 7 The Maria Grzegorzewska University, Warsaw, Poland

**Keywords:** Depression, Anxiety, Stress, Time Pressure, Wellbeing

## Abstract

**Background:**

Despite advances in technology and labour-saving devices, chronic time pressure, or the sense that you do not have enough time, is increasing globally. The implications of this for health and wellbeing are poorly understood. This study aimed to examine the impact of chronic time pressure on experiences of symptoms of depression, anxiety and stress in a European sample.

**Methods:**

A cross-sectional design was employed. A sample of 7,570 individuals, representative of the populations of the UK, Spain, Germany, Switzerland, Czechia and Poland in terms of age and gender participated. Participants completed a questionnaire containing demographic questions, the Depression, Anxiety and Stress Scale-21 to measure symptoms of depression, anxiety and stress and the Chronic Time Pressure Inventory to measure feelings of harriedness and cognitive awareness of time shortage. Hierarchical regression analysis was used to examine the predictive value of chronic time pressure on experiences of symptoms of depression, anxiety and stress.

**Results:**

Chronic time pressure was a significant predictor of symptoms of depression, anxiety and stress in all countries studied, accounting for between 5 and 24% of the variance in depression, anxiety and stress scores. Two factors of chronic time pressure differentially predicted symptoms of depression, anxiety and stress. Whilst feeling harried was universally associated with increased scores for depression, anxiety and stress, cognitive awareness of time shortage was not consistently predictive of depression, anxiety and stress.

**Conclusions:**

Chronic time pressure is associated with reduced wellbeing. Feeling chronically harried is associated with increased experiences of the symptoms of depression, anxiety and stress. The results emphasize the need for governments, employers and healthcare providers to prioritise reducing time pressure as a mechanism of improving wellbeing.

**Supplementary Information:**

The online version contains supplementary material available at 10.1186/s40359-025-03654-4.

## Introduction

Time, it’s the one thing we can never seem to get enough of, and when it’s gone, there is no getting it back. Despite significant advances in labour-saving devices and technologies [1], legislation to provide better support for working parents [2], legal protections for paid vacation [3], maximum working hours [4] and a right to disconnect [5], many people still report living in a state of chronic time pressure [6–17]. A 2018 US Gallop poll revealed that 80% of Americans report having not enough time in daily life [18]. Time pressure is thought to be rising due to the ever-increasing demands of hectic modern living [18]. Living under chronic time pressure has been associated with a broad range of negative implications for health, society and the economy [7, 18–22].

Chronic time pressure refers to the consistent feeling that there is not enough time available to complete all required tasks [7]. Chronic time pressure can be objective i.e. the actual duration of tasks exceeds the time available to complete them. It can also be subjective i.e. the persistent feeling that there is an insufficient amount of time to complete all tasks. Recent conceptualisations of chronic time pressure suggest that it has two distinct components: feeling harried and cognitive awareness of time shortage [7]. Feeling harried refers to persistent subjective feelings of apprehension, worry, anxiety and frustration arising from the experience of the perceived rapid passage of time. Cognitive awareness of time shortage refers to objective awareness of time shortage. These dimensions of time pressure can be assessed through the Chronic Time Pressure Inventory [7] which is a validated self-report measure of chronic time pressure.

Previous research has demonstrated that time pressure has the capacity to negatively impact our health, the way in which we interact with others, and how we respond to the world around us [6]. Individuals living with chronic time pressure experience reduced sleep quality [23] and have an increased likelihood of developing physical illness [24–27] including high blood pressure [28]. Critically, chronic time pressure also reduces the likelihood of engagement with health services for treatment and support risking late detection of disease and poorer treatment outcomes [29].

Chronic time pressure can result from a combination of work, social and domestic tasks. In the context of work, chronic time pressure is known to significantly impact worker wellbeing [30]. Time-related job demands are significantly correlated with job stress, with those experiencing greater time pressure at work also experiencing greater levels of stress [31] and exhaustion [32, 33]. As a result, in work, time pressure is a leading cause of burnout [34], presenteeism [35] and short- and long-term work absence [36]. The impact of job-based time pressure is not however limited to the workplace. Work-related time pressure and the financial consequences of work absence can spill over into homelife reducing the time available for family life and increasing work-to-family conflict [37]. In this context, the negative effects of time pressure appear intergenerational, with emergent evidence suggesting an association between parental time pressure and increased mental health problems amongst their children [37].

Outside of work, time pressure can result from the demands of caring for others and broader social responsibilities. In this context, women are typically found to be more time poor than men [38]. Gendered differences in time pressure appear to be driven by gender inequality in the burden of social reproductive labour during day-to-day life (e.g. child rearing, caring responsibilities and other domestic duties). As women still shoulder a disproportionate amount of care-based and domestic duties, they report greater levels of time pressure and reduced free time availability in comparison with their male counterparts [39–41].

More recently, increased use of digital technology has emerged as an additional source of time pressure. The demands of constant social connectivity coupled with a tendency for people to use digital technologies for longer than expected exacerbates feelings of time poverty [42]. These effects are compounded by the widely held belief that much of the free time people spend on digital technology (i.e. consuming social media) is a waste of time, resulting in a sense of avoidable time loss and an increase in pressure to use time more effectively in the future [42].

Whether time pressure results from work, homelife, or a combination of the two, it can have significant implications for mental health. High levels of chronic time pressure are also associated with lower wellbeing and lower life satisfaction [6]. Specifically, time pressure is associated with negative mood and reduced positive affect [18], emotional exhaustion [10, 39], and contributes to higher depression levels, particularly among working women and parents [43]. In the context of work, long working hours and time pressure are closely linked to depression and anxiety. Virtanen et al. (2011) [44] found that working over 55 h per week significantly increased the risk of mental health issues, especially in women. Greater time pressure has also been also associated with reduced wellbeing and confidence in women [12, 45], perhaps suggesting that combination of the time demands of social reproductive labour and work place them at increased risk of impaired wellbeing and quality of life.

Anxiety also correlates with increased time pressure awareness [46], with research showing that across academic [47, 48], workplace [49], and decision-making settings [50], time pressure consistently emerges as a key driver of anxiety [50, 51], particularly in younger people [52]. Time pressure can also limit our capacity to develop a positive future outlook and is therefore correlated with greater psychological distress [53]. Conversely, buying time, or making other lifestyle changes which reduce time pressure, is associated with an increase in happiness [18, 54] and improved social connectivity [55]. Being under time pressure can reduce prosocial behaviours [19] and has been shown to increase the likelihood of risky decision making in a range of contexts [55].

The negative impact of chronic time pressure on wellbeing, and the positive impact of “buying time” on happiness, suggest that our experience of time pressure may be an important precursor to the development and maintenance of feelings of depression, anxiety and stress. In particular, the persistent feeling of being harried may exacerbate feelings of worry and apprehension resulting from a perceived inability to fulfil all the required tasks in life. Similarly, consistent perceptions of failure resulting from daily demands exceeding available time might evoke persistent feelings of shame, guilt, regret and low self-efficacy.

Given high global prevalences of clinical and non-clinical depression and anxiety [56–58], and growing rates of time pressure in modern life [6], understanding the potential contribution of chronic time pressure to feelings of depression, anxiety and stress is critical to improving broader public health and reducing the burden on already strained health services. Whilst previous work has demonstrated a likely association between time pressure and mental wellbeing, studies have typically used limited sample sizes which were not representative of the population, focused singular geographic areas, and only explored time pressure in specific contexts (e.g. work). As result, it is unclear whether time pressure is a consistent determinant factor of mental wellbeing across different geographies and populations. Cross-cultural research examining the relationship between general experiences of time pressure (i.e. combining pressures from home, work and other sources) and mental wellbeing, using large representative samples is critical because time pressure experience is known to vary across cultures [59].

The current study therefore sought to explore the relationship between experiences of chronic time pressure and symptoms of depression, anxiety and stress in representative samples of the population of six European countries (UK, Spain, Switzerland, Czechia, Poland and Germany). Data was collected through the TIMED project (https://www.timed-europe.net/who-we-are) which explores the impact of digital technologies on time experience and wellbeing in the UK, Spain, Switzerland, Czechia, Poland and Germany. These countries were selected for their differing social, political, economic and gender norms, and their differing cultural profiles. Drawing data from these countries therefore enables broad comparison of the differing experiences of European citizens.

An online questionnaire was employed to measure chronic time pressure, using the Chronic Time Pressure Inventory (CTPI) [7], and symptoms of depression, anxiety and stress using the Depression, Anxiety and Stress Scale-21 (DASS-21) [60]. Participants were recruited from the UK, Spain, Switzerland, Czechia, Poland and Germany. Regression analysis was used to establish the extent to which DASS-21 scores were predicted by experiences of being harried and cognitive awareness of time shortage, gender and age. It was expected that greater experience of time pressure would be associated with female gender, and with higher scores on the depression, anxiety and stress DASS-21 subscales.

## Methods

### Participants

Participants were eligible to take part in the study if they resided in Czechia, French-speaking Switzerland, Germany, Poland, Spain or the UK, were aged 18 or above and spoke Czech, French, German, Polish, Spanish or English fluently. In total, 7570 participants completed the questionnaire composed of the measures described below (1227 in Czechia, 1230 in French-speaking Switzerland, 1225 in Germany, 1456 in Poland, 1212 in Spain and 1220 in the UK). However, 34 participants were excluded as they had provided incorrect answers to one or more of three attention check questions. Attention check questions were included to enable the research team to ensure that participants were reading and answering the questions posed, rather than just randomly responding. These included statements such as "Please select "strongly disagree" [or another specific response option, e.g. "strongly agree"] to show you are paying attention.". The final sample size therefore amounted to 7536 participants. Owing to the small number of participants who identified as a gender other than male or female, statistical analysis of data from this group of individuals was not possible. Data from these participants was therefore excluded from the analysis. Demographic characteristics of participants are shown in Table 1 and education levels are shown in Table 2.

**Table 1:** Demographic information for the sample

*Country*	*N (%)*	*Age*			*Gender*		
		Range	M	SD	Male	Female	Other gender identity or no response
*Czechia*	1212 (16.08)	18–86	45.51	15.81	605 (49.92)	599 (49.42)	8 (0.66)
*Switzerland*	1227 (16.28)	18–92	43.30	15.28	569 (46.37)	651 (53.06)	7 (0.57)
*Germany*	1225 (16.26)	18–85	45.97	15.82	604 (49.31)	615 (50.20)	6 (0.49)
*Poland*	1456 (19.32)	18–88	47.99	15.80	703 (48.28)	753 (51.72)	0 (0.00)
*Spain*	1212 (16.08)	18–82	45.01	14.73	592 (48.84)	609 (50.25)	11 (0.91)
*UK*	1204 (15.98)	18–86	46.16	16.28	596 (49.50)	600 (49.83)	8 (0.66)
*All countries*	7536 (100)	18–92	45.73	15.69	3669 (48.69)	3827 (50.78)	40 (0.53)

**Table 2:** Highest levels of education for the sample

*Country*	*N (%)* *Highest level of education*					
	Below degree level	Technical qualifications	University degree	Professional degree or equivalent	Other	Prefer not to say
*Czechia*	571 (47.11)	292 (24.09)	341 (28.14)	0 (0.00)	3 (0.25)	5 (0.41)
*Switzerland*	265 (21.60)	285 (23.23)	567 (46.21)	99 (8.07)	9 (0.73)	2 (0.16)
*Germany*	436 (35.59)	549 (44.82)	228 (18.61)	0 (0.00)	12 (0.98)	0 (0.00)
*Poland*	663 (45.54)	314 (21.57)	479 (32.90)	0 (0.00)	0 (0.00)	0 (0.00)
*Spain*	119 (9.82)	288 (23.76)	800 (66.01)	0 (0.00)	2 (0.17)	3 (0.25)
*UK*	478 (39.70)	187 (15.53)	504 (41.86)	27 (2.24)	6 (0.50)	2 (0.17)
*All countries*	2532 (33.60)	1915 (25.41)	2919 (38.73)	126 (1.67)	32 (0.42)	12 (0.16)

### Ethics

All participants gave informed consent by ticking an online form before completing the questionnaire. The study was approved by Liverpool John Moores University Research Ethics Committee (23/PSY/071). The study was conducted in adherence to the Declaration of Helsinki.

### Sampling

Stratified participant recruitment was carried out by two paid participant panel service providers: Qualtrics Panels in Czechia, Germany, Switzerland, Spain and the UK and by Pollster in Poland. These companies made the survey available to pre-screened volunteers from their panels of survey respondents comprised of the general population of Czechia, Germany, Poland, Switzerland, Spain and the UK. Participants needed to meet the following inclusion criteria to participate in the study: be aged 18 years or above, currently a resident in the country of study, and able to speak the language of study fluently. Participants who were interested in the study were then invited to complete the questionnaire. Paid panel services were used to ensure that samples were stratified to be representative of the population of each country in terms of age and gender. We aimed to recruit around 1400 participants in Poland and 1200 individuals in each of the remaining countries. Participants were rewarded by Qualtrics and Pollster for taking part in the study. Data collection commenced on the 12/01/2024 and was completed on 29/01/2024.

### Measures

Demographics: Participants indicated their gender and age.

Chronic Time Pressure Inventory (CTPI): Time pressure was measured by means of the 13-item Chronic Time Pressure Inventory [7], which assessed two dimensions of time pressure: Feeling harried (5 items) and cognitive awareness of time shortage (8 items). Feeling harried reflects a negative appraisal of being rushed, whereby concerns related to time management induce anxiety, such as worries about the effective use of one’s time. Cognitive Awareness of Time Shortage refers to the recognition of insufficient time to complete tasks or to engage in leisure activities, often accompanied by feelings of pressure to accommodate all demands. In the CTPI, these constructs are assessed through statement items, with participants indicating their level of agreement on a 5-point Likert scale, ranging from 1 (Strongly disagree) to 5 (Strongly agree). Item scores are summed to obtain the final score as well as subscale scores. An English version of the instrument was developed and validated by Denovan & Dagnall (2019) [7].

The English version was translated into Spanish, German, French and Polish by Andrew Denovan independently of this manuscript. The English version was also translated into Czech by the TIMED research team. All translations were carried out using the translation method outlined by Beaton et al. (2000) [61]. This involved the generation of forward translations from English to the new language (e.g. Czech) by two bilingual translators who worked independently. These translations were then compared, discussed and combined to produce one optimized forward translation of the measure. The optimized forward translation was then back translated (e.g. from Czech to English), and the resulting English version was reviewed and compared to the original English measure. A final version was that showed semantic and idiomatic equivalence with the original English instruments was then agreed.

Reliability of the scales was assessed using Cronbach’s Alpha. For Cognitive awareness the following alphas were observed: UK = 0.86, Switzerland = 0.83, Spain 0.82, Poland 0.86, Czechia 0.87, and Germany 0.85 indicating good reliability in all countries. For harried the following alphas were observed: UK = 0.81, Switzerland = 0.77, Spain 0.61, Poland 0.83, Czechia 0.78, and Germany 0.75.

Depression, Anxiety and Stress Scale (DASS-21) [60]: To assess common mental health problems, the 21-item DASS-21, derived from the longer 42-item DASS [62] was administered. The instrument is composed of three subscales of 7 items each, which measure levels of depression, anxiety and stress respectively. Respondents are required to indicate the extent to which 21 statements applied to them “over the past week” by selecting a response on a 4-point Likert scale ranging from 0 (Did not apply to me at all) to 3 (Applied to me very much or most of the time). Subscale scores are obtained by summing individual item scores. Higher numbers are interpreted as an indication of greater severity of depression, anxiety and stress. The DASS-21 has been widely used and was validated in non-clinical UK [62], German [63], French [64], Polish [65] and Spanish [66] samples. A Czech version of the measure was created for the study using the same translation methods employed for the CTPI. The DASS is not a diagnostic tool and therefore the analysis reported relates to the experience of symptoms of depression, anxiety and stress rather than clinical diagnoses.

Reliability of the scales was assessed using Cronbach’s Alpha. For Depression the following alphas were observed: UK = 0.93, Switzerland = 0.92, Spain 0.92, Poland 0.92, Czechia 0.92, and Germany 0.93 indicating good reliability in all countries. For anxiety the following alphas were observed: UK = 0.89, Switzerland = 0.88, Spain 0.89, Poland 0.89, Czechia 0.88, and Germany 0.87. For stress the following alphas were observed: UK = 0.90, Switzerland = 0.89, Spain 0.86, Poland 0.90, Czechia 0.88, and Germany 0.91.

### Procedure

The mean duration of study completion was 21.48 min as the survey included additional measures not reported here.

### Analysis

Data analysis was performed using SPSS version 29. Three forms of analysis were used. 1) Analysis of Variance (ANOVA) with between subjects factors of country (Czechia, Germany, Poland, Spain, Switzerland and UK) and gender (male and female), were used to explore gender and country based differences in experiences of cognitive awareness of time, harriedness, and symptoms of depression, anxiety and stress. Bonferroni post-hoc comparisons were used to explore interactions. 2) Pearson’s product moment correlations were used to establish the relationships between time pressure and symptoms of depression, anxiety and stress with time pressure in each country 3) Hierarchical multiple regression was used to explore the extent to which feeling harried and cognitive awareness of time shortage predicted symptoms of depression, anxiety and stress beyond age and gender. Hierarchical regression was selected to analyse the extent to which cognitive awareness of time pressure and harriedness were predictive of symptoms of depression, anxiety and stress beyond the demographic variables of age, gender and country. Two models were used for each analysis. Model one, explored the predictive value of age, gender and country on experiences of symptoms of depression, anxiety and stress. Model two added cognitive awareness of time pressure and feelings of harriedness to establish whether their inclusion significantly increased the predictive capacity of the model. For all analyses, the small number of people who identified as a gender other than male or female prevented inclusion of these participants in statistical analysis. The analysis therefore represents data from participants who identified as male or female.

## Results

Table 3 shows descriptive statistics for the measures of time pressure, and symptoms of depression, anxiety and stress. Examination of Table 3 indicates that females generally experienced greater levels of time pressure, and symptoms of depression, anxiety and stress than males. To explore this data further, univariate ANOVAs were used to assess the effect of gender (male and female) and country (UK, Switzerland, Spain, Poland, Czechia and Germany) on the measured variables. Table 4 shows the outcomes of this analysis.

**Table 3:** Descriptive statistics for anxiety, depression, stress, feeling harried and cognitive awareness of time shortage

*Country*	*Gender*	*Depression*		*Anxiety*		*Stress*		*Harried time pressure*		*Cognitive awareness of time pressure*	
		Mean	SD	Mean	SD	Mean	SD	Mean	SD	Mean	SD
*UK*	*Male*	5.67	5.50	3.68	4.16	5.71	4.77	2.84	.82	2.80	.73
	*Female*	7.79	6.01	6.03	5.38	8.18	5.30	3.23	.81	3.16	.74
*Switzerland*	*Male*	5.20	4.80	4.31	4.24	5.98	4.49	2.86	.81	2.98	.72
	*Female*	6.75	5.48	5.81	5.20	7.71	5.11	3.10	.83	3.11	.77
*Spain*	*Male*	5.74	4.96	4.20	4.36	7.06	4.57	3.15	.60	3.21	.65
	*Female*	6.46	5.58	5.12	5.03	8.05	4.72	3.24	.63	3.35	.69
*Poland*	*Male*	5.67	5.15	3.87	4.29	6.19	4.69	3.05	.74	3.08	.68
	*Female*	5.96	5.31	4.39	4.66	6.84	4.89	3.10	.76	3.18	.67
*Czechia*	*Male*	5.93	5.10	4.32	4.21	6.31	4.21	2.99	.75	3.15	.71
	*Female*	6.98	5.55	5.44	5.11	7.48	4.97	3.04	.77	3.19	.76
*Germany*	*Male*	5.37	5.23	3.75	4.25	5.86	4.94	2.89	.78	2.84	.75
	*Female*	6.51	5.81	4.69	4.79	7.45	5.47	3.09	.78	3.11	.78

**Table 4:** Outcomes of ANOVA analysis of cross-cultural differences in reported symptoms of depression, anxiety and stress and feelings of time pressure

*Dependent variable*	*Independent variable*	*Df*	*f*	*p*
*Harriedness*	*Gender*	1, 7495	99.97	*p* < 0.001
	*Country*	5, 7495	13.97	*p* < 0.001
	Gender * Country	5, 7495	5.80	*p* < 0.001
*Cognitive awareness of time pressure*	*Gender*	1, 7495	126.86	*p* < 0.001
	*Country*	5, 7495	36.38	*p* < 0.001
	Gender * Country	5, 7495	5.80	*p* < 0.001
*Depression*	*Gender*	1, 7495	82.24	*p* < 0.001
	*Country*	5, 7495	5.30	*p* < 0.001
	Gender * Country	5, 7495	4.50	*p* < 0.001
*Anxiety*	*Gender*	1, 7495	128.50	*p* < 0.001
	*Country*	5, 7495	8.53	*p* < 0.001
	Gender * Country	5, 7495	5.87	*p* < 0.001
*Stress*	*Gender*	1, 7495	162.66	*p* < 0.001
	*Country*	5, 7495	6.80	*p* < 0.001
	Gender * Country	5, 7495	5.60	*p* < 0.001

### Cross-cultural differences in symptoms of depression, anxiety stress and feelings of time pressure

Bonferroni post-hoc analysis of the data in Table 3 and the ANOVA analysis presented in Table 4 showed that harriedness was greater in females than males, and significantly higher in Spain than the other countries studied (*p* < 0.001). Males and females did not differ in their experience of harriedness in Czechia and Poland, but females in the UK, Switzerland, Spain and Germany experienced greater levels of harriedness than males.

Cognitive awareness of time shortage was greater in females than males and greater in Spain than all other countries, significantly lower in Poland and Czechia than all other countries. Males and females did not differ in their experience of cognitive awareness of time shortage in Czechia and Poland, but females in the UK, Switzerland, Spain and Germany experienced greater levels of cognitive awareness of time shortage than males.

Reported symptoms of depression was greater in females than males, and significantly greater in the UK than Switzerland, Poland and Germany (*p* < 0.05), and significantly greater in Czechia than Poland. Males and females did not differ in their depression scores in Poland but did in all other countries.

Reported symptoms of anxiety was greater in females than males. Anxiety was also significantly lower in Poland than all countries except Germany and Spain, and significantly lower in Germany than in the UK, Switzerland and Czechia. The difference between males and females’ anxiety scores was smaller in Poland than other countries.

Reported symptoms of stress was greater in females than males, and significantly lower in Spain than the other countries.

Table 5 shows Pearsons Product Moment correlation coefficients for the relationships between age, time pressure, and symptoms of depression, anxiety and stress. Examination of Table 3 suggests that time pressure and measures of wellbeing were negatively associated with age indicating lower levels of time pressure, and symptoms of depression, anxiety and stress with increasing age. Greater harriedness and cognitive awareness of time shortage were associated with greater reporting of symptoms of depression, anxiety and stress. These relationships were explored further using hierarchical regression. For each country hierarchical multiple regressions were conducted to assess the predictive value of age, gender, harried chronic time pressure and cognitive chronic time pressure on symptoms of depression, anxiety and stress.

**Table 5:** Correlation coefficients for the relationships between chronic time pressure, depression, anxiety and stress

	*Age*	*Stress*	*Anxiety*	*Depression*	*Harriedness*	*Cognitive Awareness of time shortage*
*Age*	-					
*Stress*	-.21**					
*Anxiety*	-.32**	.88**				
*Depression*	-.25**	.83**	.71**			
*Harriedness*	-.49**	.48**	.36**	.52**		
*Cognitive Awareness of time shortage*	-.51**	.48**	.34**	.42**	.69**	-

^**^p <.001

### Depression

Initial analysis conducted on all data revealed a significant effect of country on depression scores (*p* < 0.05), data from each country was therefore modelled separately. Model 1 and model 2 were a significant fit for depression scores in all countries. UK: M1 *F*(2, 1195) = 48.26, *p* < 0.001, M2 *F*(4, 1195) = 124.86, *p* < 0.001, Switzerland M1 *F*(2, 1219) = 50.41, *p* < 0.001, M2 *F*(4, 1219) = 117.60, *p* < 0.001. Spain M1 *F*(2, 1200) = 52.00, *p* < 0.001, M2 *F*(4, 1200) = 46.25, *p* < 0.001, Poland M1 *F*(2, 1455) = 59.35, *p* < 0.001, M2 *F*(4, 1455) = 143.93, *p* < 0.001, Czechia M1 *F*(2, 1203) = 34.76, *p* < 0.001, M2 *F*(4, 1203) = 122.91, *p* < 0.001 and Germany M1 *F*(2, 1218) = 46.26, *p* < 0.001, M2 *F*(4, 1218) = 79.40, *p* < 0.001. In all countries, there was a significant change in R^2^ from model 1 to 2 with model 2 adjusted R^2^ changes ranging from 0.05 to 0.24. Table 4 shows model outcomes. Examination of Table 4 suggests that age and CPTI Harried were significant predictors of depression in all countries, with lower age and greater experiences of harriedness being associated with an increase in depression scores.

### Anxiety

Initial analysis conducted on all data revealed a significant effect of country on anxiety scores (*p* < 0.01), data from each country was therefore modelled separately. Model 1 and Model 2 were a significant fit for anxiety scores in all countries. UK: M1 *F*(2, 1195) = 110.58, *p* < 0.001, M2 *F*(4, 1195) = 105.43, *p* < 0.001, Switzerland M1 *F*(2, 1219) = 70.84, *p* < 0.001, M2 *F*(4, 1219) = 83.36, *p* < 0.001. Spain M1 *F*(2, 1200) = 64.14, *p* < 0.001, M2 *F*(4, 1200) = 55.99, *p* < 0.001, Poland M1 *F*(2, 1453) = 41.93, *p* < 0.001, M2 *F*(4, 1453) = 87.95, *p* < 0.001, Czechia M1 *F*(2, 1203) = 42.88, *p* < 0.001, M2 *F*(4, 1203) = 76.12, *p* < 0.001 and Germany M1 *F*(2, 1218) = 67.13, *p* < 0.001, M2 *F*(4, 1218) = 73.80, *p* < 0.001. In all countries, there was a significant change in R^2^ from model 1 to 2 with model 2 adjusted R^2^ changes ranging from 0.06 to 0.14. Table 5 shows model outcomes. Examination of Table 5 suggests that age and CPTI Harried were significant predictors of anxiety in all countries, with lower age and greater experiences of harriedness being associated with an increase in anxiety scores.

### Stress

Initial analysis conducted on all data did not reveal a significant effect of country on stress scores; however, for consistency data from each country was modelled separately. Model 1 and model 2 were a significant fit for stress scores in all countries. UK: M1 *F*(2, 1195) = 104.31, *p* < 0.001, M2 *F*(4, 1195) = 133.39, *p* < 0.001, Switzerland M1 *F*(2, 1219) = 68.83, *p* < 0.001, M2 *F*(4, 1219) = 126.60, *p* < 0.001. Spain M1 *F*(2, 1200) = 70.21, *p* < 0.001, M2 *F*(4, 1200) = 86.28, *p* < 0.001, Poland M1 *F*(2, 1453) = 67.85, *p* < 0.001, M2 *F*(4, 1453) = 145.11, *p* < 0.001, Czechia M1 *F*(2, 1203) = 62.49, *p* < 0.001, M2 *F*(4, 1203) = 120.65, *p* < 0.001 and Germany M1 *F*(2, 1218) = 80.11, *p* < 0.001, M2 *F*(4, 1218) = 118.17, *p* < 0.001. In all countries, there was a significant change in R^2^ from model 1 to 2. Table 6 shows model outcomes. Examination of Table 6 suggests that age and CPTI Harried were significant predictors of stress in all countries, with lower age and greater experiences of harriedness being associated with an increase in stress scores (Tables 7 and 8).

**Table 6:** Hierarchical regression analysis for depression scores

*Model*	*Predictors*	*UK*	*Switzerland*	*Spain*	*Poland*	*Czechia*	*Germany*
*Model 1*	*R*^*2*^	.07	.08	.08	.07	.05	.07
	*Gender*	.09*	.10**	.03	-.01	.09*	.04
	*Age*	-.22**	-.24**	-.28**	-.28**	-.21**	-.26**
*Model 2*	*R*^*2*^	.29	.28	.02	.28	.29	.21
	*Gender*	.05	.06*	.36*	-.01	.08*	.02
	*Age*	-.08*	-.09**	-.23**	-.17**	-.13**	-.13**
	*CTPI Harried*	.60**	.50**	.24**	.55**	.60**	.41**
	*CTPI Awareness*	-.17**	-.02	.01	-.11**	-.18**	-.02
	*R*^*2*^* change*	.22**	.20**	.05**	.21**	.24**	.13**

^*^p <.05, **p <.001

**Table 7:** Hierarchical regression analysis for anxiety scores

Model	Predictors	UK	Switzerland	Spain	Poland	Czechia	Germany
Model 1	Adjusted R^2^	.10	.10	.10	.05	.07	.10
	Gender	.10**	.10**	.05	.03	.11**	.02
	Age	-.29**	-.29**	-.30**	-.23**	-.23**	-.31**
Model 2	Adjusted R^2^	.21	.21	.16	.19	.20	.19
	Gender	.07*	.07*	.04	.03	.10**	.01
	Age	-.18**	-.18**	-.25**	-.14**	-.16**	-.21**
	CTPI Harried	.37**	.37**	.22**	.44**	.42**	.38**
	CTPI Awareness	-.02	-.02	.05	-.09*	-.07*	-.08*
	Adjusted R^2^ change	.11**	.11**	.06**	.14**	.13**	.09**

^*^p <.05, **p <.001

**Table 8:** Hierarchical regression analysis for stress scores

*Model*	*Predictors*	*UK*	*Switzerland*	*Spain*	*Poland*	*Czechia*	*Germany*
*Model 1*	*R*^*2*^	.15	.10	.11	.08	.09	.12
	*Gender*	.11**	.12**	.06*	.03	.09*	.07*
	*Age*	-.33**	-.27**	-.31**	-.29**	-.28**	-.32**
*Model 2*	*R*^*2*^	.31	.29	.22	.28	.29	.28
	*Gender*	.06*	.08**	.04	.03	.10**	.05
	*Age*	-.17**	-.12**	-.23**	-.16**	-.18**	-.16**
	*CTPI Harried*	.41**	.36**	.21**	.44**	.45**	.33**
	*CTPI Awareness*	.04	.13**	.18**	.05	.01	.14**
	*R*^*2*^* change*	.16**	.19**	.11**	.20**	.20**	.16**

^*^p <.05, **p <.001

## Discussion

This study explored the relationship between chronic time pressure and experiences of symptoms of depression, anxiety and stress in samples representative of the populations of the UK, Spain, Germany, Switzerland, Czechia and Poland. Chronic time pressure was conceptualised as 1) a persistent feeling of being harried in daily life, and 2) persistent cognitive awareness of time shortage.

The results show that time pressure was a significant predictor of symptoms of depression, anxiety and stress in all of the countries studied. Critically, differing forms of time pressure were differentially related to experiences of the symptoms of depression, anxiety and stress. Across all countries, increased experiences of feeling harried were associated with greater experiences of the symptoms of depression, anxiety and stress, explaining between 5 and 22% percentage of the variance in DASS-21 scores beyond age and gender alone.

The relationship between cognitive awareness of time shortage and experiences of the symptoms of depression, anxiety and stress was more complex, varying by country and condition. Greater cognitive awareness of time shortage was associated with lower levels of depression symptoms in the UK, Poland and Czechia, but unrelated to experiences of depression in Spain, Germany and Switzerland. Cognitive awareness of time shortage was unrelated to experiences of anxiety in the UK, Switzerland and Spain but negatively associated with anxiety in Poland, Germany and Czechia where greater awareness of time shortage was associated with lower levels of anxiety symptoms. For stress, cognitive awareness of time shortage was predictive of stress symptoms in the Swiss, German and Spanish data, with greater awareness of time pressure being associated with greater stress.

These findings suggest that feeling chronically harried by the temporal demands of daily life may significantly contribute to poorer mental wellbeing in European citizens. In line with the hypothesis, in all countries studied, greater feelings of harriedness were associated with greater feelings of symptoms associated with depression, anxiety and stress. There are a number of ways in which time pressure may increase symptoms of depression, anxiety and stress. First, time pressure, and particularly the sense of being harried and unable to switch off from the demands of life, is associated with poorer sleep [9, 67, 68]. Critically this relationship is bidirectional in that time pressure impairs ability to achieve high quality sleep, but a lack of high-quality sleep also increases time pressure because of the impact of tiredness on subsequent functioning. A lack of time is also a barrier to improving sleep quality, because time poverty prevents individuals from implementing good sleep practices in the future [9]. Secondly, the perceived or actual failure to accomplish all tasks in the time provided may increase feelings of guilt regarding incomplete tasks, or worthlessness about the ability to cope with the time pressures of life [69]. Repeated experiences of perceived failure because of a lack of time may also exacerbate feelings of hopelessness [70]. These effects may be exacerbated by executive function being impaired by increased time pressure [71], rendering individuals with fewer cognitive resources to manage the demands of their lives and at greater risk of future task failure. Impaired executive function due to increased time pressure may also contribute to feelings of reduced concentration capacity which are often associated with feelings of depression, anxiety and stress. The collective impact of time pressure on sleep, cognition and affect therefore offers mechanisms through which time pressure contributes to the core symptomology of depression, anxiety and stress.

Whilst feeling harried was universally associated with lower wellbeing, cognitive awareness of time shortage was associated with both positive and negative outcomes in some populations. Specifically, for symptoms of depression, in the UK, Poland and Czechia, greater awareness of time shortage was associated with lower depression scores and in Poland, Czechia and Germany it was associated with lower levels of anxiety symptoms. This suggests that there may potentially be some benefit to wellbeing of being aware of time pressures. We postulate that this may reflect greater awareness enabling people to employ strategies to better manage their time, therefore reducing worry, feelings of failure and worthlessness. This suggestion is supported by previous findings that greater awareness of time demands and greater use of time management strategies can be associated with reduced experience of worry [72–74]. For stress however, greater cognitive awareness of time shortage was associated with greater feelings of the symptoms of stress, but only in Spain, Switzerland and Germany. Here we postulate that greater awareness of time shortage, but the absence of strategies to manage the shortage, may act as a catalyst for feelings of stress, with individuals who are more aware of the time pressures they face experiencing greater stress through greater awareness.

It is unclear why greater cognitive awareness of time shortage was not universally associated with increased feelings of stress and reduced feelings of depression and anxiety, in the countries studied. One possibility is that the differing local and national policies aimed at regulating working practices and providing social support moderated the effect of cognitive awareness of time shortage on mental wellbeing. However, the findings may also reflect contextual differences in the perception and experience of time pressure across the countries of study.

Time experience is known to differ on the basis of culture [59], with the temporality of a culture often being described as polychronic or monochronic. Polychronic cultures are characterised as those in which time is viewed as cyclical, flexible and adaptable. Multitasking, interruptions and flexibility around deadlines are all associated with polychronicity. This contrasts with monochronic cultures which are characterised as having a linear representation of time in which planning and deadlines are valued and adhered to. Of the countries studied, Spain’s temporal culture is more closely aligned to polychronicity whereas the others are more aligned to monochronicity. This difference in predominant temporal culture may explain when chronic time pressure had a smaller impact on measures of the symptoms of depression, anxiety and stress in Spain than in the other countries.

Although the cross-sectional nature of this study does not enable determination of whether time pressure is a causal factor in feelings of depression, anxiety and stress, it is plausible time pressure may act both as a causal factor as described above, but also as an exacerbating factor. Here, because disrupted sleep and poor concentration as a result of pre-existing depression, anxiety and stress may reduce the ability to perform tasks efficiently, resulting in greater time pressure. which in turn then increases feelings of being overwhelmed, failure, worthlessness and irritability. Reducing time pressure may therefore be one way of reducing the severity of preexisting symptoms of depression, anxiety and stress.

As in previous work, the results of this study suggest that women experience greater levels of time pressure than men [75]. Despite shifting gender roles, and greater equality in terms of parenting and employment, our findings suggest that women are still more time poor than men. Women also experienced greater levels of depression, anxiety and stress than men with gender being a consistent predictor of depression, anxiety and stress in model 1 analyses, replicating previous findings of gender inequality in wellbeing [76]. Interestingly, the inclusion of time pressure as a predictor for symptoms of depression, anxiety and stress removed or reduced the effect of gender on wellbeing. We therefore tentatively suggest that gender differences in wellbeing may in part reflect differing levels of time poverty. To fully understand the role of time pressure in wellbeing, further research is required to explore the extent to which time availability moderates the impact of other socio-demographic factors known to predict depression, anxiety and stress.

The finding that feelings of being temporally harried is predictive of symptoms of depression, anxiety and stress in six European countries suggests that time pressure may be a critical factor in the global increase in rates of depression, anxiety and stress [56–58]. This highlights the potential value to clinicians and public health officials in exploring patients’ day to day experiences of time when they present with feelings of depression, anxiety and stress. In turn, they also suggest that interventions designed to assist people to improve their time management skills [77–80] or reduce the emotional impact of time poverty [81–87] may be effective in improving wellbeing. However, given the absence of large scale assessment of the efficacy of these interventions outside of a work based context and across cultures, further research exploring their efficacy in increasing wellbeing and quality of life is therefore urgently required.

### Limitations

Paid panel services were used to ensure that the sample was representative of the population in terms of age and gender and all participants were reimbursed for participation. It is possible that the use of a paid panel service may have biased the sample because only individuals registered for the panel were eligible for participation. Critically, we did not stratify for socio-economic status, employment status and education, all of which are known to impact on time availability, depression, anxiety and stress. Future work should therefore seek to examine how these factors impact on the way in which time pressure is associated with symptoms of depression, anxiety and stress.

Relatedly, the number of people with a gender identity other than male or female was too small to enable valid statistical analysis. We are therefore unable to comment on how gender identity other than male and female may impact on the relationship between time pressure and wellbeing. As rates of depression and anxiety are known to be higher in gender minority groups than cisgender groups [88] and there is emergent evidence that the passage of time is an important factor in the lived experience of people with diverse gender identities [89], research conducted specifically on this population is required.

Whilst the current findings demonstrated differential impacts of harriedness and cognitive awareness of time pressure on symptoms of depression, anxiety and stress, these two subscales of time pressure were highly correlated with one another. Denovan & Dagnall [7] acknowledged this in their initial validation of the CPTI, however they noted that despite the correlation between both subscales, each contributed unique variance and therefore a more nuanced understanding of time pressure as a concept. A singular underlying experience of time pressure, encompassing both harriedness and cognitive awareness, could therefore explain the findings observed.

Finally, whilst reverse translation was used to ensure accurate translation of the CTPI, only the UK version was validated. The data collected for the Swiss, French, Polish, Spanish, German and Czech samples was therefore completed using unvalidated measures. Linguistic and cultural differences may have impacted the interpretation of the questionnaire in unvalidated versions ([90–92] and supplementary materials for further discussion). However participants did not report difficulty in completing the measures and Cronbach’s alpha indicated good reliability.

## Conclusions

The findings of this study demonstrate that experiences of chronic time pressure are associated with and predictive of experiences of the symptoms of depression, anxiety and stress. Specifically, greater feelings of harriedness are associated with greater experience of symptoms of depression, anxiety and stress. Whilst the precise causal mechanisms through which time pressure impacts depression, anxiety and stress remain unclear, impaired sleep, exacerbated senses of failure and hopelessness, and impaired executive functioning may be contributing factors. The results underscore the importance of considering reducing time poverty as a critical variable in achieving and maintaining wellbeing. With evidence that subjective time pressure is increasing, despite ever-advancing developments in labour-saving technologies, this work highlights the need for governments, employers and healthcare providers to act rapidly to protect citizens’ rights to time, and work towards policies which reduce the pressures on time that people experience during everyday life.

## Supplementary Information

Supplementary Material 1

Supplementary Material 2

## Data Availability

Data is provided within the supplementary information files.

## Abbreviations

CPTI: The Chronic Time Pressure Inventory, a questionnaire tool used to measure self-reported experiences of time pressure. Its two sub-scales measure 1) feeling harried and 2) cognitive awareness of time pressure
DASS-21: The short version of the Depression, Anxiety and Stress Scale which measures self-reported symptoms of depression, anxiety and stress
TIMED: The acronym for TIMe experience in Europe’s Digital age, the project through which this work was conducted
