# Supplementary material for: Chronic time pressure as a predictor of symptoms of depression, anxiety and stress
Source: BMC Psychol. 2025 Nov 24;13:1407. doi: 10.1186/s40359-025-03654-4 (PMC12751689; doi:10.1186/s40359-025-03654-4)
Supplement: Supplementary file 1 — Supplementary Material 1 [file 40359_2025_3654_MOESM1_ESM.pdf]

Discussion of the impact of linguistic differences on the findings.

Whilst reverse translation was used to ensure accurate translation of the CTPI, linguistic and cultural differences may have impacted the interpretation of the questionnaire. For example, in the Spanish language connections of the concepts of “pressure” or “urgency” with that of “time” seem to be less easily or naturally established linguistically than in English or German. This can be illustrated in two ways: The phrases *presión de tiempo* (a direct translation of *time pressure* or *Zeitdruck*) or *presión por el tiempo* (literally “pressure due to time”) are rarely encountered in oral and written language as evidenced by the low number of occurrences of the first phrase [64] and the absence of the second phrase [65] in all texts originating from Spain contained in a large corpus of the Spanish language. Rather than referring to time pressure by means of a single noun phrase equivalent to the English or German expressions, it is more common in Spanish to use descriptive or idiomatic phrases that capture elements of time pressure, such as a lack of time (*falta de tiempo*), being in a hurry (*tener prisa*) or feeling rushed (*estar apurado/a*). Another difference in the way time pressure is represented linguistically in Spanish compared to other languages can be illustrated by the translation of the phrase “running out of time”, which was translated to “quedarse sin tiempo” (literally “being left without time”) in the Spanish version of the Chronic Time Pressure Inventory. Unlike “running out of time”, which seems to convey the idea of time escaping suggesting a sense of urgency, “quedarse sin tiempo” appears to focus to a greater extent on time simply expiring, which is associated with less urgency. Given that language can influence how meaning is represented mentally [66], it is possible that Spanish speakers’ interpretation of the concept of time pressure differs from that of speakers of other languages. . This coupled with cultural differences in temporality in Spain versus the other countries of study, may explain why the predictive value of chronic time pressure was smaller in the Spanish sample than the others.
